# Newly Prepared 129Xe Nanoprobe-Based Functional Magnetic Resonance Imaging to Evaluate the Efficacy of Acupuncture on Intractable Peripheral Facial Paralysis

**DOI:** 10.1155/2022/3318223

**Published:** 2022-03-10

**Authors:** Fengyun Fan, Xiaonan Wang, Yao Lu, Kaixue Jia

**Affiliations:** ^1^Department of Acupuncture, Laishan Branch of Yantai Hospital of Traditional Chinese Medicine, Yantai 264001, Shandong, China; ^2^Department of Acupuncture and Massage, Yantai Hospital of Traditional Chinese Medicine, Yantai 264001, Shandong, China; ^3^Department of Rehabilitation, Yantai Hospital of Traditional Chinese Medicine, Yantai 264001, Shandong, China

## Abstract

This study focused on the application value of the newly prepared 129Xe nanoprobe-based functional magnetic resonance imaging (fMRI) in exploring the mechanism of the acupuncture treatment for intractable facial paralysis, expected to provide a theoretical reference for the mechanism of acupuncture for the treatment of facial paralysis. In this study, 30 patients with intractable peripheral facial paralysis (experimental group) and 30 healthy volunteers (control group) were selected. All patients were scanned by the newly prepared 129Xe nanoprobe-based fMRI technology, and then brain functional status data and rating data were collected. fMRI scanning results showed that multiple brain regions were activated in the experimental group before treatment, among which the central posterior brain, insula, and thalamus were positively activated, while the precuneus, superior frontal gyrus, and other parts showed signal reduction. After treatment, several brain regions also showed signal enhancement. Comparisons within the healthy control group also showed activation in multiple brain regions, including the lenticular nucleus, inferior frontal gyrus, and superior temporal gyrus, while in the experimental group, no signal changes were detected in these brain regions. At the same time, comparison of fMRI images of patients with intractable peripheral facial paralysis before and after treatment showed that the cerebellar amygdala, superior frontal gyrus, cerebellar mountaintop, and other brain areas were activated, and all showed positive activation. After treatment, the average House–Brackmann (H-B) and Sunnybrook scores of the experimental group were 3.82 and 51, respectively, and the change was significant compared with that before treatment (*P* < 0.05). In conclusion, the newly prepared 129Xe nanoprobe-based fMRI scan can reflect the functional changes of cerebral cortex after acupuncture. The acupuncture treatment may achieve its therapeutic effect by promoting the functional reorganization of the cerebral cortex in the treatment of intractable facial paralysis.

## 1. Introduction

Facial paralysis, also known as facial nerve palsy, has a rapid onset and is mainly manifested by paralysis of lateral facial expressions, disappearance of forehead lines, crooked mouth and eyes, and inability to close eye clefts and frown [1]. Facial paralysis can be divided into peripheral facial paralysis and central facial paralysis according to the different places of injury. Peripheral facial paralysis, also known as bell palsy or facial neuritis, mainly manifests as lesions in the facial nerve and facial nerve nucleus. It is an infectious disease arising from the virus lurking in the facial nerve sensory ganglion and associated with neurogenic injury, facial nerve vascular oppression, facial nerve inflammation, or ear-related diseases [2–4]. Peripheral facial paralysis is extremely common in clinical practice, mostly occurring on one side, and can occur at any age, but those aged 20–40 are predominantly affected, and the number of male patients is more than females [5–7]. Intractable peripheral facial paralysis can be regarded as the sequelae of peripheral facial paralysis, including Hunt syndrome and Bell paralysis [8–10].

The number of patients suffering from intractable peripheral facial paralysis is increasing in recent years, and its incidence is closely related to several factors. First, the level of facial nerve injury has a far-reaching influence on the treatment of facial paralysis. An extremely high degree of facial nerve injury indicates that the treatment of facial paralysis becomes more difficult and the probability of cure is reduced. Worse still, it often develops into intractable facial paralysis. Secondly, the delayed introduction of acupuncture and moxibustion also increases the difficulty of treatment later. In addition, the climate has an impact on the rehabilitation of facial paralysis. In spring and summer, the treatment of facial paralysis requires longer, the treatment effect is poor, and the patient is difficult to recover. Finally, refractory facial paralysis often occurs in people with underlying diseases or low immunity, including people with hypertension, diabetes, old age, and infirm moods and depression [11–15]. Studies have shown that about 65% of people with facial paralysis are also depressed [16–18]. In the treatment of facial paralysis, it is recommended to use glucocorticoids and various antiviral drugs in the early stage, while external direct medication, taking neurotrophic agents, neurological rehabilitation, and surgical decompression also have a positive impact on the treatment and recovery of facial paralysis [19–21]. As a traditional Chinese medicine treatment, acupuncture has a long history. According to Huang Di Nei Jing Su Wen written one thousand years ago, the ancients often used acupuncture to treat peripheral facial paralysis. Such treatment is simple and reduces the oppression on the nerves and inflammatory oedema. It is safe and effective, with no drug intervention and no side effects and thus has been widely promoted [22]. Facial paralysis is a predominant disease treated by acupuncture and moxibustion, and it has been identified by the World Health Organization as one of the diseases that acupuncture and moxibustion are suitable in the early stages.

However, the mechanism of action is still unclear. In recent years, with the rapid development of imaging technology, functional magnetic resonance imaging (fMRI) has been widely used in the treatment of intractable peripheral facial paralysis using acupuncture. fMRI can visualize the functional activities of human tissue and organs through information data, facilitate direct analysis, and is noninvasive, noncontact, and nondamage [23–25]. The specific action principle of fMRI is as follows: blood oxygen level dependent (BOLD) is used to observe blood flow and blood volume in some functional areas of the human brain. When functional areas are activated, blood flow increases and the oxygen metabolism value will increase, thus increasing oxygenated hemoglobin and decreasing deoxyhemoglobin in this area [26–28]. The sensitivity of the newly prepared 129Xe nanoprobe is more than 10,000 times higher than that of the traditional proton, and its application in fMRI measurement of intractable peripheral facial paralysis can enhance the accuracy of data [29].

In the study, 60 subjects were selected and divided into two groups: intractable peripheral facial paralysis group and healthy control group, with 30 patients in each group. Intractable peripheral facial paralysis patients were treated with acupuncture, and fMRI scans were performed before and after treatment, while only one fMRI scan was performed for each in the healthy control group, and data of brain functional status of the two groups were collected, respectively. Meanwhile, Sunnybrook facial nerve assessment data and House–Brackmann (H-B) functional grading data of the two groups were collected for analysis. The study aimed at exploring the application value of newly prepared 129Xe nanoprobe-based fMRI in exploring the mechanism of the acupuncture treatment for intractable peripheral facial paralysis, expected to provide a theoretical reference for the mechanism of acupuncture treatment of facial paralysis.

## 2. Materials and Methods

### 2.1. Research Subjects

In this study, 30 patients with intractable peripheral facial paralysis aged 20–59 who were admitted to hospital from January 10, 2019, to May 10, 2021, were selected and defined as the experimental group. At the same time, 30 healthy volunteers were selected as the healthy control group. There were 16 male and 14 female patients in the experimental group with an age range of 20–59 years old and an average age of 47.83 years old. There were 15 male and 15 female volunteers in the control group with an age range of 21–58 years old and an average age of 46.72 years old. 30 intractable peripheral facial paralysis patients were scanned by newly prepared 129Xe nanoprobe-based fMRI technology before and after acupuncture treatment, while each in the healthy control group only needed one fMRI scan. This study has been approved by the Ethics Committee of the hospital, and the family members of the patients were informed of this study and signed the informed consent.

Inclusion criteria for intractable peripheral facial paralysis patients: (I) diagnosed with intractable peripheral facial paralysis according to the diagnostic criteria of traditional Chinese and Western medicine; (II) patients who signed the informed consent; (III) female patients not in their menstrual stage and pregnancy stage; (IV) those not suffering from serious organ diseases, hereditary diseases, or neurological diseases; (V) conventional MRI intracranial scan showed no abnormal condition; and (VI) patients without contraindications for fMRI examinations.

Exclusion criteria: (I) patients not diagnosed as intractable peripheral facial paralysis; (II) people with severe allergic constitution; (III) patients with other serious underlying diseases; (IV) female patients during menstruation or pregnancy; (Vv) patients with a history of mental illness; and (VI) patients younger than 20 years old.

Criteria for termination and exclusion: (I) patients who cannot normally complete fMRI scanning; (II) patients who did not cooperate in the follow-up after treatment for index evaluation; and (III) patients with severe intracranial lesions.

The inclusion criteria for the healthy control group were the same as the abovementioned criteria II–VI for the refractory intractable peripheral facial paralysis group, and the exclusion criteria satisfied II–VI. The criteria for termination and exclusion were the same as those mentioned above.

### 2.2. Treatment Plan

The dicang, Yangbai, Hegu, Yangbai, Neiting, and quanliao were chosen as the main points; the auxiliary points included Fengchi, Touwei, Sizhukong, Cuanzhu, Chengjiang, Qianzheng, Renzhong, and Yifeng. Professional acupuncturists performed the operation, and the stimulation intensity should be based on the patient's moderate perception. The needle was retained for 30 minutes each time, and the acupuncture was performed 3 times per 1 w. A total of 15 times were required for a course of treatment.

### 2.3. Process of Acupuncture

First, 75% alcohol was used for skin disinfection. The sterile stainless-steel needle (0.35 mm × 40 mm) was used to needle the patient at the Hegu point. It was completed by a professional acupuncturist to avoid the error due to the technique strength difference. When the needle depth reached 15 mm, the even reinforcing reducing method was used to twirl the needle (angle <90°), slight lifting, and inserting (amplitude <3 mm), and the frequency was kept at a speed of no more than 60 times/min. fMRI scan data were collected after Deqi.

### 2.4. fMRI Scan Settings

**Figure 1:**
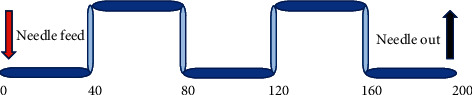
Cross-acquisition data diagram of the resting state and task state.

fMRI data of intractable peripheral facial paralysis patients were collected before and after treatment, while fMRI data of volunteers in the healthy control group only needed to be collected once. The fMRI scanning time was set to 30 minutes. The functional images of patients in resting state and acupuncture state were collected. fMRI data of resting state were collected for 10 min. fMRI data of acupuncture state were collected according to the following settings. After the fMRI scanning at the resting state was completed, the fMRI data at acupuncture state were collected immediately after Deqi, as shown in Figure 1.

The superconducting magnetic resonance scanner equipped with the newly prepared 129Xe nanoprobe was used to scan for six sequences, including functional brain imaging sequences at resting state and acupuncture state, as well as location image, T2W1 image, T1-weighted 2D anatomical image, and T1-weighted 3D anatomical image. The sequence parameters at resting state are set as follows: 36 layers, field of view (FOV) 250 mm × 250 mm, repetition time (TR) 3000 ms, echo time (TE) 30 ms, slice (SL) 3 mm, and flip angle (FA) 90°. The layer spacing is 1 mm, and the resolution is 64 × 64. The parameter setting at acupuncture state is the same as that in rest state.

### 2.5. Observation Indicators

For intractable peripheral facial paralysis patients, the scores of the Sunnybrook facial nerve rating scale and H-B functional grading of the facial nerve before and after treatment are recorded. The score range of the Sunnybrook scale is 0–100, mainly assessed from the morphological changes of the eyes (blepharoplasty), face, mouth, and expressions. A score closer to 100 indicates a better facial nerve function. The H-B functional grading score is mainly assessed from the changes in facial appearance and the tension of facial muscles at rest and during movement. It is divided into I–VI grades. A higher grade indicates worse facial nerve function of the patient.

### 2.6. Statistical Methods

SPSS software was used for statistical analysis of the data. Data conforming to normal distribution were expressed as mean ± standard deviation (*x* ± *s*). *t* test was used to represent measurement data, and *χ*^2^ test was used to represent count data. *P* < 0.05 indicated a statistical difference.

## 3. Results

### 3.1. fMRI Data

**Figure 2:**
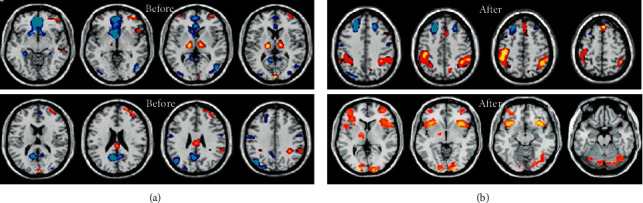
fMRI images of patients with intractable peripheral facial paralysis: (a) before acupuncture and (b) after acupuncture.

fMRI images of acupuncture state at Hegu point and resting state of patients with intractable peripheral facial paralysis before and after treatment were collected, as shown in Figure 2.

### 3.2. Activation of Brain Regions of Patients with Intrinsic Peripheral Facial Paralysis

**Figure 3:**
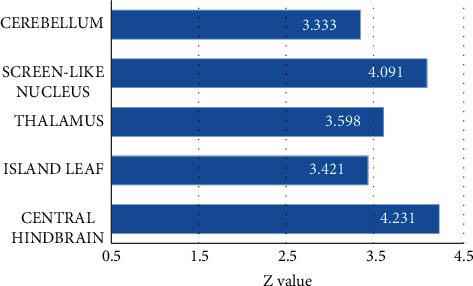
Positive activation of brain regions of patients with intractable peripheral facial paralysis before treatment.

**Figure 4:**
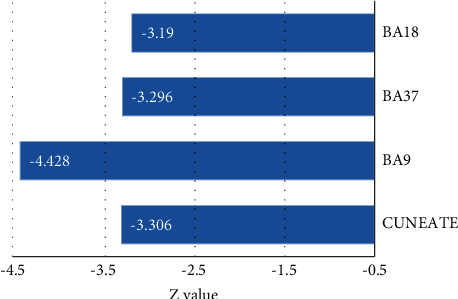
Negative activation of brain regions of patients with intractable peripheral facial paralysis before treatment. BA18 was the lingual gyrus, BA37 was the fusiform gyrus, and BA9 was the superior frontal gyrus.

**Figure 5:**
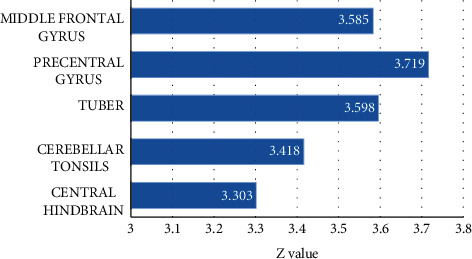
Activation in various brain regions of patients with intractable peripheral facial paralysis after treatment.

Patients with intrinsic peripheral facial paralysis received acupuncture at Hegu before and after treatment, and fMRI images at task state were collected. The intragroup comparison results before treatment showed that multiple brain regions were activated, among which the central posterior brain, insula lobe, and thalamus were positively activated, while the precuneus, superior frontal gyrus, and other parts showed a signal reduction response. After treatment, multiple brain regions also showed signal enhancement, as shown in Figures 3–5.

### 3.3. fMRI Images in the Healthy Group and Experimental Group

**Figure 6:**
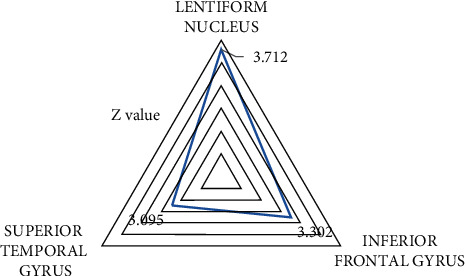
Activation of brain regions after acupuncture in healthy volunteers.

Healthy volunteers were acupunctured at the Hegu point and scanned by fMRI to obtain task-state images. Intragroup comparisons showed that multiple brain regions were activated, including the pectin nucleus, inferior frontal gyrus, and superior temporal gyrus, as shown in Figure 6. Compared with the control group, no positive activation signal was detected in the lenticular nucleus, inferior frontal gyrus, and superior temporal gyrus before treatment in the experimental group.

### 3.4. fMRI Images before and after Treatment

**Figure 7:**
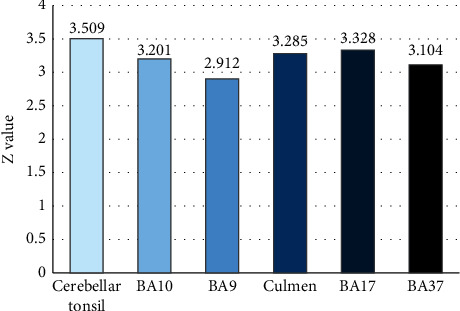
Activation of brain regions in patients with intractable peripheral facial paralysis before and after treatment. BA9 and 10 were superior frontal gyrus, BA17 was inferior occipital gyrus, and BA37 was fusiform gyrus.

Comparison of fMRI images of intractable peripheral facial paralysis patients before and after treatment showed that multiple brain areas were activated, and all showed positive activation, including cerebellar tonsil, superior frontal gyrus, and cerebellar mountaintop, as shown in Figure 7.

### 3.5. Sunnybrook Facial Nerve Assessment Results

**Figure 8:**
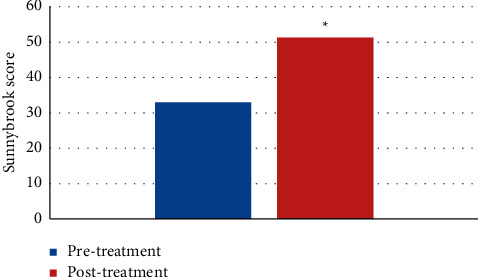
Sunnybrook facial nerve assessment results of patients with intractable peripheral facial paralysis before and after treatment. *∗* represented a significant difference, *P* < 0.05.

Intractable peripheral facial paralysis patients were evaluated for Sunnybrook facial nerve before and after treatment. The Sunnybrook score of the experimental group was 32.928 points before treatment, and 51.283 points on average after treatment. The results showed that the score after treatment was significantly higher than that of the patient before treatment (*P* < 0.05), as shown in Figure 8.

### 3.6. Results of H-E Function Classification

**Figure 9:**
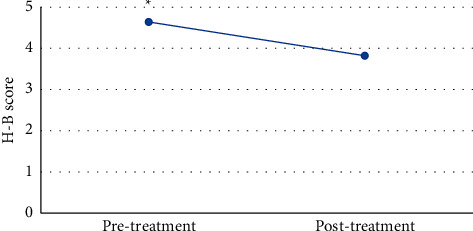
H-B scores of patients with intractable peripheral facial paralysis before and after treatment. *∗* represented a significant difference, *P* < 0.05.

Intractable peripheral facial paralysis patients were evaluated by H-E functional grading before and after treatment. The average H-E grade of patients with intractable facial paralysis in the experimental group was 4.64 before treatment and 3.82 after treatment. The results showed that the score after treatment was significantly lower than that before treatment (*P* < 0.05), as shown in Figure 9.

## 4. Discussion

At present, intractable peripheral facial paralysis, as a common and frequent disease, has attracted widespread attention. Traditional Chinese medicine believes that wind chill, as an external factor, often invades facial meridians, blocking qi and blood at the invaded site and causing the mouth and eyes to be skewed. At the same time, lack of qi and blood and physical weakness are regarded as internal causes, which result in blocked meridians and facial paralysis, together with external causes. Additionally, modern medicine explains that the pathogeny of this disease is acute oedema arising from facial nerve compression or inflammation, and that common pathological changes include lost myelin sheath, swelling, and oedema. According to research statistics, about one to four cases per 10,000 children have facial palsy [30]. There are a variety of methods for the treatment of peripheral facial paralysis, including scraping, cupping, fumigation, acupuncture, and acupoint embedding, among which acupuncture is widely adopted because of the advantages of less points and strong stimulation of the meridians. Efficacy evaluation of intractable peripheral facial paralysis requires a unified evaluation standard, so as to facilitate the evaluation of the effectiveness of acupuncture in different studies. The H-B evaluation standard and the Sunnybrook facial nerve evaluation system are often applied in many studies. The H-B evaluation standard is the unified evaluation standard adopted by the Department of Otolaryngology and Head and Neck Surgery in the United States, which is characterized by a simple operation and a high repetition rate. The Sunnybrook facial nerve evaluation system, developed by Canadian researchers, is widely used in various efficacy evaluation studies due to its effectiveness and high sensitivity [31–33]. In this study, the newly prepared 129Xe nanoprobe-based fMRI scanning technology was used, and the two evaluation criteria were used as the auxiliary methods to discuss the application value of fMRI in exploring the mechanism of the acupuncture treatment for intractable peripheral facial paralysis. The study found that, after acupuncture treatment, the H-B score was significantly lower than that before treatment (*P* < 0.05), and the Sunnybrook facial nerve evaluation system score was significantly higher than that before treatment (*P* < 0.05), indicating that the acupuncture treatment achieved good effects in the treatment of intractable peripheral facial paralysis patients.

In this study, 30 patients with intractable peripheral facial paralysis and 30 healthy volunteers were selected as the research subjects, defined as the experimental group and the healthy control group, respectively. fMRI scanning was performed before and after treatment for the patients, while fMRI scanning was only performed once for the healthy control group. Data of brain functional state of the two groups were collected, respectively. Comparison of fMRI images at task state of intractable peripheral facial paralysis patients before and after treatment showed that multiple brain areas were activated and all showed positive activation, including the cerebellar tonsil, superior frontal gyrus, and cerebellar mountaintop. This indicated that the acupuncture treatment achieved good results by promoting the reorganization of these cortical functions, and these areas are relevant to cognition and emotion functions, which have indications for the mechanism of acupuncture treatment for intractable peripheral facial paralysis. Several researchers applied fMRI technology in the treatment of intractable peripheral facial paralysis by acupuncture and found there was functional reorganization of the cerebral cortex, which was consistent with our results [34]. It was reported that after multiple fMRI scans of a Bell palsy patient, it was found that the functions of several brain regions of the patient changed from disconnection to enhanced connection after treatment, which also indicated that the mechanism of acupuncture treatment of facial paralysis was closely related to the functional reorganization of the cerebral cortex [35]. In addition, through multiple fMRI scans of patients with intractable peripheral facial paralysis at different stages, researchers found that the cerebral cortex responded differently to acupuncture at the Hegu point. It once again proved that the functional state of cerebral cortex plays a key role in the mechanism of acupuncture in the treatment of facial paralysis [36].

## 5. Conclusion

In the study, the fMRI images at task state of intractable peripheral facial paralysis patients before and after treatment showed that multiple brain areas were activated and all showed positive activation, including the cerebellar tonsil, superior frontal gyrus, and cerebellar mountaintop. At the same time, the H-B score increased and the Sunnybrook score decreased significantly after treatment, which further verified the good therapeutic effect of acupuncture, suggesting that acupuncture treatment for refractory facial paralysis can achieve good therapeutic effects by promoting the reorganization of these cortical functions. However, some limitations in the study should be noted. The sample size is small, which will reduce the power of the study. In the follow-up, an expanded sample size is necessary to strengthen the findings of the study. In conclusion, this study reveals that the newly prepared 129Xe nanoprobe-based fMRI scanning technology plays an important role in exploring the mechanism of acupuncture in the treatment of refractory facial paralysis. In future research, the specific location of acupuncture treatment for intractable peripheral paralysis can be further explored to provide a theoretical reference for the mechanism of acupuncture treatment of facial paralysis.

## Data Availability

The data used to support the findings of this study are available from the corresponding author upon request.
